# Dogs (*Canis familiaris*), but Not Chimpanzees (*Pan troglodytes*), Understand Imperative Pointing

**DOI:** 10.1371/journal.pone.0030913

**Published:** 2012-02-08

**Authors:** Katharina C. Kirchhofer, Felizitas Zimmermann, Juliane Kaminski, Michael Tomasello

**Affiliations:** Max Planck Institute for Evolutionary Anthropology, Leipzig, Germany; University of Western Ontario, Canada

## Abstract

Chimpanzees routinely follow the gaze of humans to outside targets. However, in most studies using object choice they fail to use communicative gestures (e.g. pointing) to find hidden food. Chimpanzees' failure to do this may be due to several difficulties with this paradigm. They may, for example, misinterpret the gesture as referring to the opaque cup instead of the hidden food. Or perhaps they do not understand informative communicative intentions. In contrast, dogs seem to be skilful in using human communicative cues in the context of finding food, but as of yet there is not much data showing whether they also use pointing in the context of finding non-food objects. Here we directly compare chimpanzees' (N = 20) and dogs' (N = 32) skills in using a communicative gesture directed at a visible object out of reach of the human but within reach of the subject. Pairs of objects were placed in view of and behind the subjects. The task was to retrieve the object the experimenter wanted. To indicate which one she desired, the experimenter pointed imperatively to it and directly rewarded the subject for handing over the correct one. While dogs performed well on this task, chimpanzees failed to identify the referent. Implications for great apes' and dogs' understanding of human communicative intentions are discussed.

## Introduction

Much recent research has found that chimpanzees understand the goals and even intentions of others [1]. However, many studies have also found that chimpanzees have difficulties using a human's referential gesture (e.g. pointing) to locate hidden food [2]. Of course, if given enough trials, chimpanzees can learn to use the pointing gesture, and they find it easier to learn this when the pointing finger is close to the target location, i.e. within 5 cm – perhaps due to local enhancement [3]. Chimpanzees raised by humans may be better able to learn human gestures as well [4]–[6].

The problem is not that chimpanzees do not follow the gaze direction of humans to outside targets; they *do* do this [7]–[8]. If that target is food, then they may go and fetch it. However, in the so-called object choice task in which the food is hidden, the situation is different. Here the human points to one of several opaque containers. In this situation the subject must not only locate the target but also infer *why* the pointer is directing attention to the container, which in itself is uninteresting. Human infants as young as 14 months are successful in this task [9].

Perhaps surprisingly, domestic dogs are skilled in using a variety of communicative cues, including pointing, in object choice tasks [10]. Their performance cannot be explained by learning during the experiment as in many studies they demonstrate such skill from the very first trial. Also their performance cannot be explained by major learning during ontogeny as puppies from an early age seem to use human communication flexibly [11]–[13]. It is more likely that dogs' skills with human communication are an adaptation to life with humans and are influenced by selection processes during domestication. This is also supported by the fact that untrained wolves perform poorly [12], [13]–[14]. Even though wolves can learn how to use pointing after receiving special training, e.g. clicker training [15]–[16], dogs develop this skill earlier and need no specific training in order to follow pointing [14].

There are several aspects of the object choice task, as it is typically administered, that may make it more problematic for chimpanzees than for dogs. The first is that typically for the chimpanzees the containers and the food are on the human's side of some caging or barrier. This makes the task artificial in the sense that the human does not really need the chimpanzee's help in locating the food – she could easily just lift the containers and look herself. For the dogs the containers are not behind any barrier but are freely accessible to them, which may make the setting more natural and it may be easier for them to understand and attend to the relevance of the communicative gesture. Furthermore, dogs are mostly tested in a more distal set-up, where they have to move towards one of the referents, while primates are mostly positioned within reaching distance of the referents [17]. A second issue is that chimpanzees might follow the pointing gesture to the container and assume that the human intends to indicate the container itself, not what might be inside. In this referential, communicative setting the chimpanzees may simply follow gaze to an object, whereas dogs may always expect to find something interesting when following pointing.

Finally, the human's pointing gesture in the object choice task is informative pointing, in which the goal of the pointer is to help the recipient altruistically by providing information useful to her – specifically, the location of hidden food. Dogs' domestication may have led them to assume cooperation from humans. Chimpanzees may make no such assumption – leading to the possibility that chimpanzees would be much more successful in a task in which the human's communicative motive was more directive or imperative.

In the current study, therefore, we presented both chimpanzees and domestic dogs with a modified object choice task. In the task the human pointed to one of two objects she desired, with both potential referents visible, out of reach of the human, and located some distance from the subject. In addition, the motive of the pointing gesture was clearly directive (imperative), as the human did not point to food for the subject's benefit but to an object she wanted the subject to bring to her.

## Methods

We compared two groups of chimpanzees (*Pan troglodytes*) in their ability to use an imperative pointing gesture to infer the target object in a modified object choice task. In order to verify the method and examine whether it generally worked we compared the behaviour of the chimpanzees with that of a sample of domestic dogs (*Canis familiaris*). In the current setting a human communicator pointed to one of two objects with the respective referents visible and in reach of the subjects. Objects were placed behind subjects, out of their direct grasp. Handing over the referent to the communicator led to a direct reward for the subject. Therefore, the communication was highly relevant for the subject to help find the correct object. For practical reasons, the studies of the chimpanzees and the dogs were conducted separately, with four different experimenters (diploma student SK for the group of chimpanzees housed in Leipzig Zoo, co-author JK or a keeper for the group of chimpanzees on Ngamba Island, assistant KS for the dogs) and in different physical settings. We used exactly the same methods whenever possible.

This study adhered to the “Guidelines for the Use of Animals in Research”. IRB approval was not necessary for this kind of study because no special permission for use of animals (chimpanzees and dogs) in socio-cognitive studies is required in Germany. All proccedures were performed in full accordance with German legal regulations and the guidelines for the treatment of animals in behavioural research and teaching of the Association for the Study of Animal Behaviour (ASAB). For the chimpanzees on Ngamba Island (Uganda) animal husbandry and research complied with the“PASA Primate Veterinary Healthcare Manual” and the “Chimpanzee Sanctuary & Wildlife Conservation Trust Policy”.

All dogs were registered in the dog database of the Department of Developmental and Comparative Psychology (MPI EVA) and recruited by phone. All dog owners with their dogs participated on a voluntary basis.

### Subjects

#### Chimpanzees

We pre-tested 23 chimpanzees (*Pan troglodytes*), of which three had to be excluded from the study because during the warm-up phase they did not fetch any objects in their area upon request. Therefore the analysis includes data from 20 chimpanzees (11 males, 9 females). Eleven individuals (four males, seven females, age range: 4–32 years, five nursery-reared, six mother-reared) were housed at the Wolfgang Köhler Primate Research Center at Leipzig Zoo (Germany). The nursery-raised chimpanzees were reared from a young age with peer conspecifics and a good deal of contact with humans and their artefacts, but without human training aimed at specific behavioural outcomes, that is, they were not trained to perform certain “human-like” activities. Nine chimpanzees (seven males, two females, age range: 7–24 years, all reared by humans from a certain point in their lives but living in constant contact with conspecifics) were from the Ngamba Island Sanctuary, Uganda. All chimpanzees lived with conspecifics in social groups. Chimpanzees were pre-selected based on their motivation to fetch objects, which was information given by the keepers, who commonly incorporate the fetching of objects from the cage into the animals' routine (this includes fetching only one or one out of several objects). The 11 chimpanzees from the Leipzig group were selected out of 14 chimpanzees, while the 9 chimpanzees from the Ngamba Island group were selected out of 44 chimpanzees.

In Leipzig, the apes were housed in semi-natural indoor (overall 533 m2) and outdoor (4000 m2) enclosures with regular feedings, enrichment and water ad lib. Subjects participated in the study voluntarily and were deprived of neither food nor water. In Ngamba, the apes were allowed to roam freely on the 40-ha island during the day and spent the night in seven interconnected sleeping rooms (overall 140 m2) with regular feedings and water ad lib. Subjects participated in the study voluntarily and were deprived of neither food nor water.

For the present study, subjects were tested individually. If mothers had young infants, they were not separated. Subjects had previously participated or were currently participating in other studies.

#### Dogs

We tested 32 dogs (14 males, 18 females, age range: 1–10 years). Like the chimpanzees, we aimed at pre-selecting dogs based on their motivation to fetch objects. This was done by briefly interviewing the dog owners over the phone, before the dogs were invited to take part in the study. However, even though there was an attempt to pre-select, thirty-four dogs did not pass the warm-up phase, as they were not interested in retrieving stationary objects. Seven dogs passed the warm-up phase but had to be excluded at the very beginning of the experimental phase as they lost interest, which means that, for instance, they could not be motivated anymore to fetch any objects. Dog subjects were all family-owned dogs of different breeds, recruited over a database at the Max Planck Institute for Evolutionary Anthropology (see Table S2 for more information on sex, age and breed). Some of the dogs had participated in other studies, though it was ensured that none of the dogs had ever participated in a study which included fetching objects upon receiving a communicative cue.

### Set-up and Materials

#### Chimpanzees

**Figure 1:**
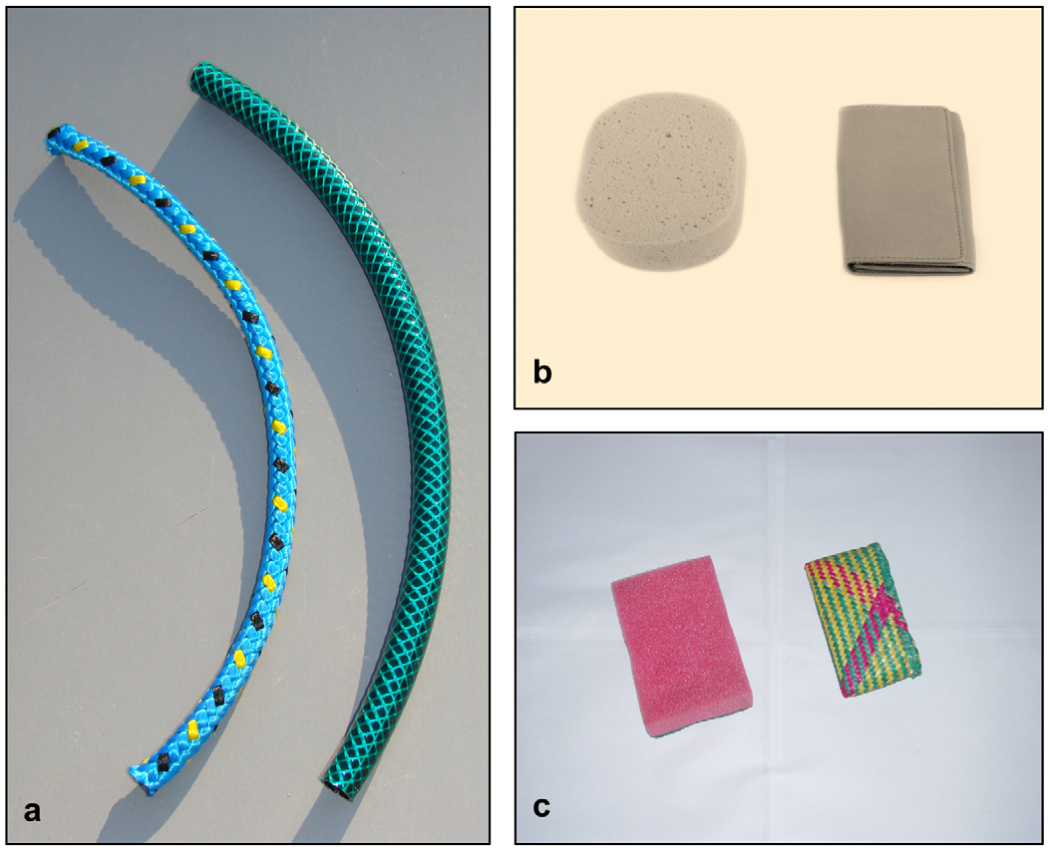
Examples for object pairs. Rope and hose (a) were used with Leipzig chimpanzees, sponge and leather case (b) with the dogs, sponge and bast case (c) with Ngamba chimpanzees.

Subjects were presented with pairs of everyday objects commonly used by humans, which were similar in certain features but never identical (see Figure 1). The chimpanzees in Leipzig and the dogs were presented with four different pairs of objects while the chimpanzees in Uganda were presented with eight different pairs of objects. One reason for this difference for the Ngamba Island chimpanzees was that they would develop string preferences for objects quickly and we wanted to ensure that the possibility that they develop a preference for one object over the other was reduced to a minimum.

**Figure 2:**
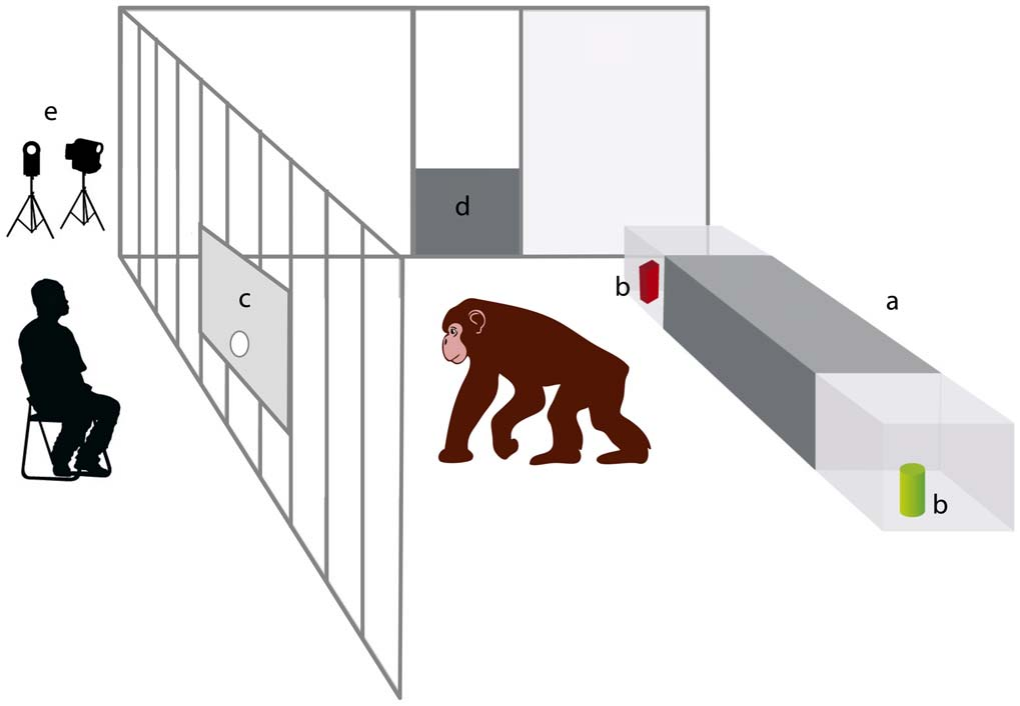
Experimental set-up for the Leipzig chimpanzees. (a) container, (b) objects, (c) testing window, (d) hydraulic door, and (e) camera.

The general setting was identical for all subjects. Two objects were presented such that they were within reach for the subject but not for the human. The rooms differed in some respects for the Leipzig and the Ngamba chimpanzees, which is why the setting had to be adapted accordingly. The testing room in Leipzig comprised an ape and a human area, separated by transparent Plexiglas or mesh panels. The pairs of objects were presented in a rectangular Plexiglas container (147×20×30 cm) with two separate chambers at the distal ends (distance: 1.10 m). It was mounted in the back part of the apes' area at a distance of 1.70 m from the subject (see Figure 2). In the container's resting position, the two chambers were covered by a Plexiglas board. To gain access to one of the chambers the board had to be pushed in one direction, which at the same time blocked the other chamber, such that the choice was unambiguous and no second choice was possible. Subjects could pass the objects out through the hole (diameter: 6 cm) in the centre of a Plexiglas window, upon which they received a reward.

On Ngamba Island subjects were tested in an empty room. The two objects were presented on a board (with a distance of 1.50 m between them) which was placed outside the testing room close to the bars such that subjects could reach through the bars to get an object. The experimenter was also located outside the testing room but on the other side, i.e. opposite the side with the board. So after taking an object subjects had to turn around to bring it to the experimenter, to whom they could hand it through the metal bars. To make sure that subjects could not reach for the second object after making a choice, an assistant who stood behind the board stepped forward and immediately pulled it away. The assistant was always located between and equidistant to both objects. At no time did the assistant gaze at or look in the direction of either of the two objects. Instead the assistant gazed at the opposite side of the setting, fixing upon an imaginary spot.

#### Dogs

The setting for the dogs was identical to the setting used with the Ngamba chimpanzees. One major difference between dog and ape settings is often that while there is a barrier between ape and human, there is normally no such barrier in studies with dogs. To see whether such a barrier between the human and the subject would affect dogs' behaviour, half of the dogs were tested with a barrier between dog and human and the other half without this barrier. The objects (see Figure 1) were presented on a board, which was placed outside the testing arena behind a fence. The objects were presented with a distance of 1.50 m between them. The experimenter was located opposite the board. So after taking an object subjects had to turn around to bring it to the experimenter, to whom they could give it. Again, like with the chimpanzees, to make sure that subjects could not reach for the second object after making a choice, the assistant stepped forward and immediately pulled the board away and behind a fence out of the subjects' reach.

The pointing gesture, the accompanying gaze and the vocal command were exactly the same for both groups of chimpanzees and the dogs. The experimenter was positioned equidistant between the two objects during each trial. All trials were videotaped.

### Procedure

The study comprised two different phases: a warm-up phase and an experimental phase. In the warm-up phase, which was conducted to familiarize subjects with the general procedure, the assistant placed only one object (which was later not used in the test) in the container or on the board and subjects were repeatedly encouraged by the experimenter to retrieve this object and to give it to her. For the chimpanzee group in Leipzig, before each warm-up trial the assistant placed the object in the container while the subject was waiting in the adjacent room. Then the experimenter entered and the subject was released into the testing compartment. The assistant then centred the subject by offering fruit juice or peanuts for the chimpanzees or a piece of dry dog food for the dogs and as soon as the subject looked up from drinking/eating and was paying attention to the experimenter, the experimenter started requesting the object. For the Ngamba group and the dogs, before each warm-up trial the assistant placed the object on the board while the subject stayed with the experimenter, who constantly offered food.

To request the object during the warm-up the experimenter showed a reward (a grape or peanuts for the chimpanzees or a piece of dry dog food for the dogs) to the subject and then indicated that she wanted the object by beckoning (repeatedly opening and closing her hand) and commanding “Give it to me!” with a stern tone of voice and a serious facial expression. No directional cues such as gaze alternation or pointing were used. We did not have to train chimpanzees to exchange objects for food as they were used to exchanging things with the keepers, e.g. when something had fallen into their cage.

When the subject handed over the object she received the reward and the experimenter expressed her pleasure at receiving the object. If the subject did not deliver the object for over 1 min the experimenter offered a second piece of reward. If the subject did not react for another minute the experimenter acted frustrated, threw the reward back into the bucket and left. The Leipzig chimpanzees and the dogs received four warm-up trials, while the Ngamba chimpanzees, who always went to get the object immediately upon request, received only two warm-up trials. Subjects who exchanged the object in three out of the four trials (or in both trials for the Ngamba chimpanzees) were moved to the experimental phase. As in Leipzig the warm-up also served to familiarize subjects with the Plexiglas box, the location of the object was counterbalanced between the left and right compartment, while for the Ngamba chimpanzees and for the dogs the object was presented in the middle of the platform.

After the warm-up phase, subjects entered the experimental phase. Experimental trials were identical to the warm-up trials with the exception that the assistant placed two objects into the container/onto the board (always placing the left object first and continuing with the right one), such that there was a distracting object and a target object. The experimenter then indicated which one she wanted by explicitly pointing to it. The gesture was accompanied by the same vocal cues as in the warm-up phase. Pointing was conducted with the extended index finger of the ipsilateral arm. The distance between the index finger and the target object was approximately 2.40 m. The experimenter continued gesturing until the subject had decided on one of the two objects. Every pointing gesture was accompanied by gaze alternation (with raised eyebrows) between the subject and the object. During each pointing gesture the experimenter gaze alternated approximately three times before producing the gesture again. If the subject took the target object this was regarded as a choice and the experimenter started beckoning for it. If the subject handed over the target object, she received the reward. Whenever the subject chose the distractor or took the target object but did not hand it over within 2 min (from the first pointing gesture) the experimenter showed signs of frustration and discarded the reward. If the subject could not have seen the pointing cue, e.g. because she took an object before being focused (i.e. before drinking juice), the trial was repeated at the end of the session or at the beginning of the next session, which then took place on a different day. If the subject did not take an object within 2 min or showed signs of distress, the session ended immediately and was repeated on a different day.

Subjects received four sessions of four trials (chimpanzees in Leipzig) or two sessions of eight trials (chimpanzees on Ngamba and dogs), totalling 16 trials in each case. The break between sessions was a minimum of one day and a maximum of six days. Each pair of objects was used once or twice per session, with the order of pairs randomly determined for each session. The location of the target object was semi-randomized and counterbalanced within each session with the stipulation that the target could not be in the same location in more than two consecutive trials. Each object of each pair was the target object in half of the trials. For the chimpanzees in Leipzig the experimenter was a person who was not completely new to the subjects but had no special relationship with them. To see if relationship had an effect, we had two different people communicate with the subjects on Ngamba Island. In one session the experimenter was a stranger and in the other it was one of the keepers with whom they were very familiar. Four subjects started with the stranger as the experimenter, the other five started with the keeper as the experimenter. Dogs were tested by a person who was a complete stranger.

### Data scoring and analysis

The subjects' responses were coded live by the respective experimenter and completed from the videotapes. We scored whether the subject retrieved the target and also whether she gave this to the experimenter. A second coder independently coded 100% of the Leipzig chimpanzee data, 20% of the Ngamba chimpanzee data and 20% of the dog data. These 20% were randomly chosen. Interobserver reliability was perfect for both chimpanzee groups (Cohen's Kappa: κ = 1) and excellent for the dogs (Cohen's Kappa κ = 0.93). For statistical analysis we performed binomial tests, separately for each subject. The expected proportion of correct choices was 0.5. We then compared the number of correct choices of the chimpanzees with that of the dogs using an independent-samples t-test We also compared the dogs that were tested with a barrier to the dogs that were not, using an independent-samples t-test. We also compared each species separately against chance performance, using one-sample t-tests. We further analyzed whether subjects learned over trials by using a repeated measures ANOVA, comparing the first half of trials with the second half of trials, with species as a between-subject factor. Finally we analyzed the chimpanzees separately to see if the respective group (Leipzig vs. Ngamba) had an effect, using an independent-sample t-test and also compared the number of correct choices of chimpanzees in the Ngamba group when tested with a stranger compared to when tested with a familiar person, using a paired-sample t-test. All statistical tests were two-tailed. Levene's test of equality of variances revealed that for all comparisons equal variance could be assumed. We checked whether the assumptions for ANOVA were fulfilled by visually inspecting plots of residuals versus expected values. This did not indicate any obvious violations of the assumptions. The data were normally distributed according to a Kolmogorov-Smirnov Test.

## Results

For the chimpanzee group, no subject chose the target object significantly differently from chance level. This is despite the fact that they were motivated to hand over the selected object, which they did in 95.56% of the trials. For the dog group, 9 of the 32 subjects chose the target referent at above-chance levels (see Table S1 and S2 for individual data).

**Figure 3:**
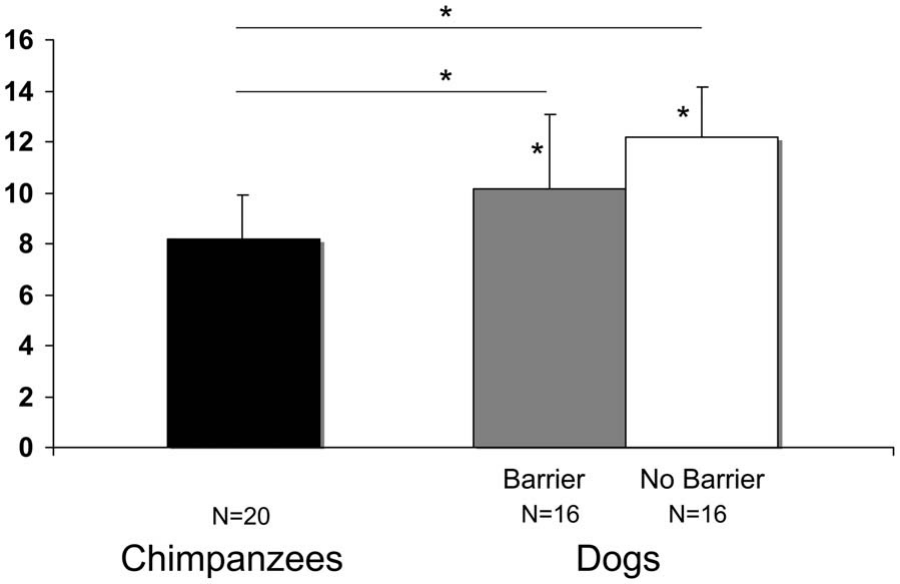
Decision behaviour. Mean number of trials in which subjects from the different species retrieved the correct object (+STD). Asterisks indicate results different from chance (*p*<0.05).

A comparison with chance for each species separately showed that the chimpanzees did not choose the target significantly differently from chance levels (M = 8.15, SD = 1.79, t(19) = 0.38, p = 0.711), while the dogs chose the target above chance levels (M = 11.16, SD = 2.65, t(31) = 6.73, p<0.0001). We then further checked whether the barrier between the human and the dog affected dogs' behaviour, which it did because without a barrier dogs were significantly more successful (without barrier: M = 12.18, SD = 1.94, with barrier: M = 10.13, SD = 2.92, t(30) = 2.354, p = 0.025). Even though the group with a barrier performed above chance on its own (t(15) = 2.913, p = 0.011), we only compared the group of dogs which had a barrier between them and the human to the chimpanzees. This comparison showed that dogs chose the target significantly more often than the chimpanzees (t(34) = 2.38, p = 0.026) (Figure 3).

To see if any learning took place we compared the first half of trials with the last half of trials using a 2-way ANOVA. The main factor of order had no effect (F(1,50) = 1.033, p = 0.31), and there was no interaction with species (F(1,50) = 2.41, p = 0.127). However, as there was a trend for the interaction we compared the first half of trials with the second half of trials for each species separately. There was no effect for the chimpanzees (t(19) = 1.37, p = 0.186) or the dogs (t(31) = 0.502, p = 0.62). To exclude the possibility that results were influenced by the fact that subjects lost interest during the trials and sessions, first trial data were analyzed. First trial data showed that only 9 out of 20 chimpanzees chose the target object in the first trial (binomial test p = 0.82) but 24 out of 32 dogs chose the target object in the first trial (binomial p = 0.007). A comparison of chimpanzees and the dog group tested with a barrier showed that in their first trial there was a significant difference between both species (Fisher's exact test: p = 0.04). To see if the performance of subjects generally varied among sessions (data was separated into 4 sessions with 4 trials each) we conducted an ANOVA with species as the between-subjects factor and session as the within-subjects factor. Session had no main effect (F(3,150) = 0.493, p = 0.68) and there was also no interaction with species (F(3,150) = 2.031, p = 0.11). Finally, we looked at the chimpanzee data separately. A comparison of both groups (Leipzig vs. Ngamba) revealed that there was no effect of group (Leipzig: M = 8.36, SD = 1.29, Ngamba Island: M = 7.89, SD = 2.32, t(18) = 0.58, p = 0.57). We also analyzed the data from the chimpanzees on Ngamba Island separately to see whether relationship with theexperimenter had an effect. A direct comparison showed that subjects did not choose differently when they were with a stranger or a familiar person (t(8) = 1.46, p = 0.184). Subjects did not prefer the target above chance when the experimenter was a stranger (M = 3.44, SD = 1.42, t(8) = 1.17, p = 0.276) or a person with whom they were very familiar (M = 4.44, SD = 1.67, t(8) = 0.8, p = 0.447). Finally we analyzed whether rearing history (nursery-reared vs. mother-reared) had an effect on the performance of the chimpanzees. This was not the case (nursery: M = 7.93, SD = 1.94, mother: M = 8.67, SD = 1.36, t(18) = 0.84, p = 0.412).

## Discussion

In the current study, as in previous studies, dogs outperformed chimpanzees in an object choice task. The fact that the dogs succeeded in the current task shows that a species which is generally skilled when it comes to using human social cues, has no problem solving this task. The new finding here is that this remains true even if the target referent is not hidden food but an object which is not interesting in itself. The comparison made here is in some sense fairer since in previous studies comparing chimpanzees and dogs, the dogs were allowed to move freely while the chimpanzees were seated behind a Plexiglas panel with the cups containing the food being located on the human's side. In order to make a choice, the chimpanzees in other studies had to stick their finger through a hole in a panel, which may have unnecessarily restricted their behaviour [13], [18]. How details of the procedure can affect subjects' behaviour (and therefore species comparisons) is shown by the fact that dogs were a little less successful, though still above chance performance, when there was a barrier between them and the human. However, comparing dogs and chimpanzees, which had exactly the same setting, still shows an effect of species. The current results thus add to the growing body of literature which shows that chimpanzees seem to have difficulties in using a human's pointing gesture, while dogs seem to have no problem with this. Also, the difference between dogs and chimpanzees is apparent from the very first trial, showing that it cannot be explained by learning during the study.

Even though the general context was much more natural than in previous studies, chimpanzees still failed the task. This means that their difficulties in previous object choice tasks cannot be due to ambivalent reference (e.g. the cup instead of the reward in the cup) as in the current study no inference about absent objects was required. In addition, the current setting provides a more natural communicative circumstance – the human needed the chimpanzee to fetch something inside her cage and she communicated imperatively. Furthermore, the attention of the subjects was focused as much as possible on the pointing gesture, as the referents were not located on the same visual plane as the cue and there was some cost of moving toward the distal referents. Note that chimpanzees understood the general idea of the procedure as they did hand over one of the two objects but the communicative gesture did not help them to make a correct choice. In addition, chimpanzees' failure to interpret the referential aspect of the pointing gesture seems to be independent of the relationship to the experimenter and also independent of their rearing history. However, it could be that even though the human communicated imperatively, the chimpanzees struggled with the still generally cooperative nature of the task. There is evidence that chimpanzees may perform better if the communicative gesture is presented in a more competitive context [19] or it is used to indicate which cup *not* to choose [20]. This may be because in competitive contexts, signals coming from the human suddenly become more relevant [21].

This current finding would seem to be discrepant with chimpanzees' documented skills of gaze-following to outside targets. However, gaze-following can be seen as a kind of exploitative behaviour in which one individual simply uses the gaze direction of another – even if that other is unaware – to search for interesting things. In the object choice task, the experimenter clearly intends things toward the chimpanzee recipient, and they presumably know this at some level. In addition, the chimpanzees have a current goal of getting the food. In this situation, chimpanzees do not use a pointing gesture, accompanied by gaze, to a specific target – even though in the current studies they only had to follow the point to a visible object. Somehow, the communicative context and their own current goals made this into a too difficult-to-comprehend situation for the chimpanzees, that is, more than a simple gaze-following situation.

Most fundamentally, these results suggest that understanding intentions is one thing, but understanding communicative intentions is another. In particular, in Tomasello's (2008) analysis, comprehending an act of referential communication involves understanding two levels of intentions [22]. First, the comprehender must understand that the experimenter intends for her to attend to a particular referent. Then, the comprehender must try to figure out why the experimenter wants her to attend to that referent – is it to inform her of something helpfully? Or perhaps the experimenter wants something for herself. Chimpanzees' general failures in the object choice task suggest that they do not process these two levels of referential communication readily. Their failures in the task presented here in particular suggest that in this situation, where the processing of two levels is important, they even fail to comprehend the referential intention itself – or else they do comprehend it, but they do not think it important for their current goal of getting the food.

The results presented here are in contrast to very recent findings by Mulcahy and Call (2009), who found that chimpanzees did better in an object choice task if the choices were far apart. The authors argue that the cost of moving in the more distal task puts pressure on the subject to attend to the cue. This suggestion would be difficult to reconcile with our findings as here the subjects also have to move in order to fetch one object. More likely, we believe, is that Mulcahy and Call's set-up rendered the referential aspect of the gesture as well as its specificity unnecessary. While attending to one cup, the other was no longer in view, which made deciphering the reference unnecessary. All the subject had to do was move to the left or right cage (a procedure probably enhanced by the daily routine of zoo-keeping) and then the subject found a cup. In the current study, both objects were in the subject's view as soon as she turned around to get one of the objects.

In any case, the current results help us to specify in much more detail which aspects of communication in the object choice task are difficult for chimpanzees. Given that they are presumably one of the animal species that is cognitively most similar to humans – and humans comprehend communicative intentions in the object choice task from as early as 14 months of age, i.e. prelinguistically [9] – it is important to identify their difficulties in detail. It also raises the question of why species which do not struggle with this task, for example domestic dogs, are so skilful. Dogs' readiness to use human communicative cues seems to be a special adaptation to life with humans and the result of certain selection pressures during domestication [11], [13]–[14], [23]. Dogs' special receptiveness to human cooperative communication makes them the perfect social tool for certain activities like herding, hunting etc. [24]. Which mechanism best explains dogs' use of human communication is still an open question. One hypothesis is that dogs see human communication as imperatives and spatial directives, ordering to them what to do and where to go next [24]–[26].

## Supporting Information

Table S1 **Number of correct choices (out of 16) for each chimpanzee.**
(DOC)

Table S2 **Number of correct choices (out of 16) for each dog.**
(DOC)
