# Supplementary material for: Dogs (Canis familiaris), but Not Chimpanzees (Pan troglodytes), Understand Imperative Pointing
Source: PLoS One. 2012 Feb 8;7(2):e30913. doi: 10.1371/journal.pone.0030913 (PMC3275610; doi:10.1371/journal.pone.0030913)
Supplement: Table S1 — Number of correct choices (out of 16) for each chimpanzee. (DOC) [file pone.0030913.s001.doc]

Table S1

*Number of correct choices (out of 16) for each chimpanzee.*

| **Subject** | **Group** | **Age (years)** | **Sex** | **No. correct** | | |
| --- | --- | --- | --- | --- | --- | --- |
|  |  |  |  | **Sum of trials** | **First half of trials** | **Second half of trials** |
| Frodo^a^ | Leipzig | 13 | M | 10 | 7 | 3 |
| Fraukje^b^ | Leipzig | 31 | F | 7 | 5 | 2 |
| Dorien^a,b^ | Leipzig | 27 | F | 10 | 7 | 3 |
| Sandra^a^ | Leipzig | 14 | F | 10 | 6 | 4 |
| Lome | Leipzig | 5 | M | 9 | 5 | 4 |
| Robert^b^ | Leipzig | 32 | M | 8 | 3 | 5 |
| Ulla^b^ | Leipzig | 30 | F | 8 | 5 | 3 |
| Natascha^a,b^ | Leipzig | 27 | F | 7 | 5 | 2 |
| Patrick^a^ | Leipzig | 10 | M | 7 | 2 | 5 |
| Pia^a^ | Leipzig | 7 | F | 7 | 3 | 4 |
| Taï | Leipzig | 4 | F | 9 | 6 | 3 |
| Asega^b^ | Ngamba | 11 | M | 7 | 4 | 3 |
| Baluku^b^ | Ngamba | 11 | M | 8 | 3 | 5 |
| Indi^b^ | Ngamba | 10 | M | 8 | 4 | 4 |
| Kidogo^b^ | Ngamba | 25 | F | 5 | 3 | 2 |
| Mawa^b^ | Ngamba | 13 | M | 8 | 2 | 6 |
| Okech^b^ | Ngamba | 8 | M | 11 | 6 | 5 |
| Tumbo^b^ | Ngamba | 20 | M | 10 | 7 | 3 |
| Umutama^b^ | Ngamba | 13 | M | 4 | 2 | 2 |
| Yoyo^b^ | Ngamba | 10 | F | 10 | 4 | 6 |

* Indicates data significantly different from chance (binomial test, expected proportion of correct choices = 0.5, p < 0.05).

^a^ These chimpanzee subjects had also participated in the study of Mulcahy and Call (2009).

^b^ These chimpanzee subjects were nursery reared for some time in their life.
