# Supplementary material for: Dogs (Canis familiaris), but Not Chimpanzees (Pan troglodytes), Understand Imperative Pointing
Source: PLoS One. 2012 Feb 8;7(2):e30913. doi: 10.1371/journal.pone.0030913 (PMC3275610; doi:10.1371/journal.pone.0030913)
Supplement: Table S2 — Number of correct choices (out of 16) for each dog. (DOC) [file pone.0030913.s002.doc]

Table S2

*Number of correct choices (out of 16) for each dog.*

| **Subject** | **Breed** | **Group** | **Age (years)** | **Sex** | **No. correct** | | |
| --- | --- | --- | --- | --- | --- | --- | --- |
|  |  |  |  |  | **Sum of trials** | **First half of trials** | **Second half of trials** |
| Pine | German Shorthaired Pointer | Leipzig | 7 | F | **14*** | **8*** | 6 |
| Arthos | Labrador Retriever | Leipzig | 9 | M | 12 | 5 | 7 |
| Cara | Labrador Retriever | Leipzig | 12 | F | **14*** | 6 | **8*** |
| Chima | Rhodesian Ridgeback | Leipzig | 8 | M | 10 | 4 | 6 |
| Taira | Labrador R. x Mongrel | Leipzig | 3 | F | 12 | 7 | 5 |
| Luck | Great Dane x Mongrel | Leipzig | 6 | M | **15*** | 7 | **8*** |
| Maya | Labrador Retriever | Leipzig | 5 | F | 12 | 4 | **8*** |
| Amy | Golden Retriever | Leipzig | 7 | F | **13*** | 5 | **8*** |
| Zhadan | Golden Retriever | Leipzig | 6 | M | 11 | 5 | 6 |
| Shanti | Rottweiler | Leipzig | 2 | F | 10* | 5 | 5 |
| Luna | German Shepherd x Mongrel | Leipzig | 9 | F | 9 | 4 | 5 |
| Quincy | German shepherd | Leipzig | 10 | M | **13*** | **8*** | 5 |
| Amur | Hovawart x Mongrel | Leipzig | 3 | M | **16*** | **8*** | **8*** |
| Ambula | Rhodesian Ridgeback | Leipzig | 8 | M | 12 | 7 | 5 |
| Jimmy | Labrador Retriever | Leipzig | 9 | M | 10 | 5 | 5 |
| Baghira | German Shepherd x Mongrel | Leipzig | 5 | F | 12 | 4 | **8*** |
| Bärbel | Belgian shepherd | Leipzig | 5 | F | 10 | 6 | 4 |
| Biene | Labrador | Leipzig | 6 | F | 8 | 4 | 4 |
| Cora | Labrador | Leipzig | 4 | F | 12 | 6 | 6 |
| Emily | Labrador | Leipzig | 8 | F | 12 | 6 | 6 |
| Fenja | Giant Schnauzer | Leipzig | 2 | F | 8 | 4 | 4 |
| Fynn | Australian Shepherd | Leipzig | 2 | M | 8 | 4 | 4 |
| Gerda | Poodle x Labrador | Leipzig | 2 | F | **16*** | **8*** | **8*** |
| Gonzo | Labrador | Leipzig | 6 | M | 10 | 6 | 4 |
| Karah | Labrador | Leipzig | 8 | F | 8 | 4 | 4 |
| Kimi | Labrador | Leipzig | 3 | F | 10 | 6 | 4 |
| Maxl | Harzer Fuchs | Leipzig | 2 | M | 7 | 3 | 4 |
| Pepe | Weimaraner | Leipzig | 4 | M | **16*** | **8*** | **8*** |
| Punk | German Shepherd x Mongrel | Leipzig | 9 | M | 9 | 4 | 5 |
| Qooper | Border Collie | Leipzig | 1 | M | **13*** | 7 | 6 |
| Scully | Border Collie | Leipzig | 3 | F | 7 | 3 | 4 |
| Sydney | Border Collie | Leipzig | 10 | F | 8 | 5 | 3 |

* Indicates data significantly different from chance (binomial test, expected proportion of correct choices = 0.5, p < 0.05).
